# Informatics for the Planet: Integrating Species, Ecosystems, and Geophysical Data to Manage the Health of Earth Systems

**DOI:** 10.2196/113778

**Published:** 2026-11-03

**Authors:** Kathleen Gray, Arriel Benis, Sune Dueholm Müller, Kenneth Winkel, Ann Borda

**Affiliations:** 1 Centre for Digital Transformation of Health, Faculty of Medicine, Dentistry and Health Sciences, The University of Melbourne, Level 8, Melbourne Connect Building, 700 Swanston Street, Melbourne, Victoria, 3053, Australia; 2 Department of Biomedical Engineering, Pratt School of Engineering, Duke University, 1427 Fitzpatrick Center, Box 90281, Durham, NC, 27708, United States, 1 (919) 660-5131; 3 Department of Biomedical Informatics, Jacobs School of Medicine and Biomedical Sciences, University at Buffalo, State University of New York, Buffalo, NY, United States; 4 Department of Informatics, Faculty of Mathematics and Natural Sciences, University of Oslo, Oslo, Norway; 5 Melbourne School of Public Health, Faculty of Medicine, Dentistry and Health Sciences, University of Melbourne, Melbourne, Australia; 6 The Alan Turing Institute, British Library, London, United Kingdom

**Keywords:** planetary health informatics, medical informatics, planetary health, digital determinants of health, sustainable health care, sustainability, climate health

## Abstract

Biomedical and health informatics has traditionally focused on human-centric clinical data to support individual patient care and health systems. However, contemporary health challenges driven by climate change, biodiversity loss, and ecological degradation demand an expanded paradigm that addresses interdependencies among human, animal, and environmental health. This editorial introduces a new *JMIR Medical Informatics* section called “Planetary Health Informatics”—an essential evolution extending informatics praxis beyond human health care and toward Earth’s interconnected systems. *JMIR Medical Informatics* is launching a call for papers for this new section dedicated to advancing planetary health informatics (PHI) theory, methods, and applications for a sustainable future shaped by humans’ impact on Earth. We invite authors to submit work that explores how PHI integrates species, ecosystem, and geophysical data to address critical global challenges, including metagenomic disease surveillance, climate-related hazard forecasting, environmental pollution monitoring, and health infrastructure decarbonization. By synthesizing broader ecological frameworks, we present a road map for transforming fragmented data streams into planetary-scale intelligence.

## Introduction

Biomedical and health informatics (BMHI) has been driven by health care professionals’ desire for more systematic management of patient information to support better clinical decision-making [1]. Most recently, the BMHI clinical trajectory has been accelerated by the convergence of the Internet of Things, social media, and AI [2].

At the same time, the evolution of BMHI has been molded by an expanding recognition of health determinants that extend well beyond the social domain to encompass environmental, ecological, and digital factors [3,4]. Frameworks such as Hancock’s Mandala of Health, which situates human health directly within the biosphere [5], and Dahlgren and Whitehead’s socio-environmental model [6,7] highlight the deep interplay between planetary health ecosystems and community well-being, while emerging paradigms increasingly identify digital determinants as critical drivers of health equity and access [8]. As a bridge between these complex dimensions, integrative frameworks, such as One Digital Health (ODH), have emerged, offering a systematic approach to harmonizing digital health innovations across human, animal, and environmental ecosystems [9]. These conceptual shifts converge with the rise of personal and community empowerment narratives, the expansion of supranational technology corporations, and the globalization of knowledge and resource flows. In this context, BMHI has become the most authoritative source of scientific research and professional practice knowledge for guiding digital innovation and governing information infrastructure in human health systems and services [10,11].

As it converges with the science and technology of bioinformatics and biomedical engineering [12,13] and becomes more explicit about its value at the level of the global communities [14,15], BMHI faces a profound challenge: how to engage with, adapt to, and incorporate the more-than-biomedical and more-than-human dimensions of health [16]. Health information systems that do not support the integration and interpretation of all relevant data for the stability of life on Earth have diminishing value. It is already clear that some information technology “solutions” in health care and beyond are unsustainable and are inadvertently destabilizing the planet [17].

Consequently, the management of conventional digital health data, patient information, and clinical knowledge must become more ecologically sophisticated. It must reflect our emerging understanding of observable, measurable interrelationships among the health of humans, animals, and other life-forms and the stability, extent, and availability of the collective biochemical and geophysical resources that form a planetary system of interconnected ecosystems [18,19].

## From Human-Centric Systems to Planetary Perspectives

While a holistic perspective on health, care, and well-being has long been a characteristic of indigenous human cultures [20], the contemporary concept of “planetary health” only took shape in the 1980s before gaining widespread global prominence through the 2015 Rockefeller Foundation–Lancet Commission [21]. Notably, 2015 marked a watershed year for global sustainability frameworks, coinciding with the adoption of the United Nations (UN) Sustainable Development Goals [22], the Paris Climate Agreement [23], and the Sendai Framework for Disaster Risk Reduction [24]. Within this evolving policy landscape, planetary health distinguished itself from related paradigms, namely Ecohealth (rooted in environmental science) [25] and One Health (rooted in veterinary medicine), by placing human medicine at its core [26,27]. Nevertheless, all three frameworks remain deeply aligned around the critical nexus connecting human, animal, and ecosystem health.

Planetary health informatics (PHI), therefore, represents a critical advance in BMHI. It demands a wider vision and praxis, extending the continuum beyond human-centric bench research, bedside care, and public health and toward care for the Earth’s interdependent systems. The need to coordinate and connect a wider range of digital information systems and platforms to support decision-making for planetary health is already recognized in principle [28,29], particularly in human-centric health information systems [30,31]. However, informatics has much more work to do to enable an inclusive, scalable view of the health of all life-forms and the endless cycling of geochemical and hydrological resources throughout the environment [32].

Some familiar examples suggest how important this work can be. One instance is the legacy of the COVID-19 pandemic [6]. The global scramble for information to curb the pandemic’s spread highlighted a multiplicity of approaches to managing relevant data and multidimensional ways of understanding the vulnerability and resilience of populations [33,34]. This is reiterated in the current highly pathogenic avian influenza H5N1 outbreak [35]. Another example is the proliferation of personal mobile sensing devices that generate large volumes of data about individuals’ lifestyles and health. Data capture is often designed to support personal benchmarking or friendly competition, but it is almost never designed to prompt collective insights into social or environmental issues [36,37]. A third example, which is now attracting much attention, is the rapidly spreading use of AI to support decision-making in health care. Optimistic investments in health applications of AI are offset by deep concerns about AI’s potential environmental impacts, human rights violations, and other ethical issues, as well as problematic regulatory stances across geographies [38-40].

## Informatics Priorities for Earth’s Interconnected Systems

However, much of the informatics work needed to support science-based planetary health decision-making is unfamiliar to the discipline. For example, wetlands are a crucial resource and a nature-based solution at the intersection of public and planetary health. Perhaps the first planetary health treaty—the Ramsar Convention on Wetlands established in 1971, which predates the Rio Conventions—created clear opportunities and needs for integrated data management. Yet, over 50 years later, only about half of the convention’s signatories maintain and share national wetland inventories, with datasets on wetland status and diversity, as well as food resources, carbon sequestration potential, water flow and availability, and animal migration patterns [41]. Furthermore, current efforts to harmonize essential data for understanding wetlands and other aspects of geodiversity have not accounted for medicinal resources and the regulation of diseases and pests, suggesting insufficient engagement from the human health sector in this work [42,43].

BMHI can respond to the urgency of treating the climate and nature crisis as one indivisible global health emergency, as flagged by coordinated international medical journal editors [30]. It can contribute to resilience and remediation in the Anthropocene by coevolving with both the informatics of animal health [44] and, going much further, the knowledge and practice found in environmental and community information systems [45,46]. Indeed, signs exist that such new digital One Health research and development collaborations can flourish [47].

One example is a pilot project that linked electronic health records for humans and companion animals in shared local environments. This work emphasized the need for stronger interinstitutional and interprofessional collaborations to facilitate information system interoperability and make it feasible to investigate human-animal-environment health interactions on a global scale [48]. This study cited the Monarch Initiative as an exemplar of the type of platform needed to enable cross-species health data integration at this ambitious scale [49].

Another area of emerging work is managing indigenous or traditional local knowledge more respectfully to more sustainably govern social and ecological systems across sectors and scales [50]. To avoid instrumental, anthropocentric decision-making about people-nature relationships, a typology commissioned by the Intergovernmental Platform on Biodiversity and Ecosystem Services characterizes health as a cross-cutting value in different worldviews and knowledge systems [51].

Moreover, BMHI researchers and practitioners are developing frameworks for planning and implementing planetary health information systems. One such example proposes aligning informatics work with the UN Sustainable Development Goals [52]. Another, more elaborate example of such work is the ODH framework [9], which is actively being tested and adapted for cross-sectoral pathogen tracking and infrastructure analysis in resource-constrained environments [11]. These frameworks shift the focus of informatics away from the one-way integration of data and decision-making for human health care as we know it and toward imagining a planetary-scale learning health system [53,54].

Informatics can continue to refine these frameworks, drawing on the multidisciplinary analysis of major planetary health system considerations [55-58] to transform relevant data into careful decisions across health information systems, such as the following:

- Environmental, ecological, and biodiversity monitoring, conservation, and preservation systems: These include systems for managing threats to the stability of cultures, populations, and microbiomes via metagenomic surveillance and by tracking the loss of local traditional medicines and resistance to antimicrobial drugs [59], as well as full, open access digital archives of major biodiversity collections, such as those of Kew Botanical Gardens [60,61].
- Weather and climate adaptation systems: These include systems for predicting and managing climate-related health hazards (eg, the spread of vector-borne diseases, heat waves, floods, and storms) [62] and using digitized biodiversity collections, satellite technology, and environmental sensors (eg, e-DNA) to detect and monitor coastal hazards (eg, algal and jellyfish blooms) [63,64].
- Air, fresh water, ocean, and soil quality systems: These include systems for managing pollution events and pervasive contaminants in contexts such as nanoplastics, e-waste, and nuclear waste [65,66].
- Systems for a circular economy and sustainability: These include systems for managing major health and care sectors sustainably in contexts such as carbon footprinting and life-cycle assessments of service delivery and funding models, equipment and supply chains, and physical infrastructure investment [17,61,65,67].

We argue that BMHI has a key contribution to make for improving planetary health outcomes by envisioning a more holistic digital transformation of health care and developing methods and tools for the systematic, shared integration of data, information, and knowledge flows for interspecies and ecosystem health.

The complex interdependencies between Earth systems and human health, which define the scope of PHI, are synthesized in Figure 1.

**Figure 1.**
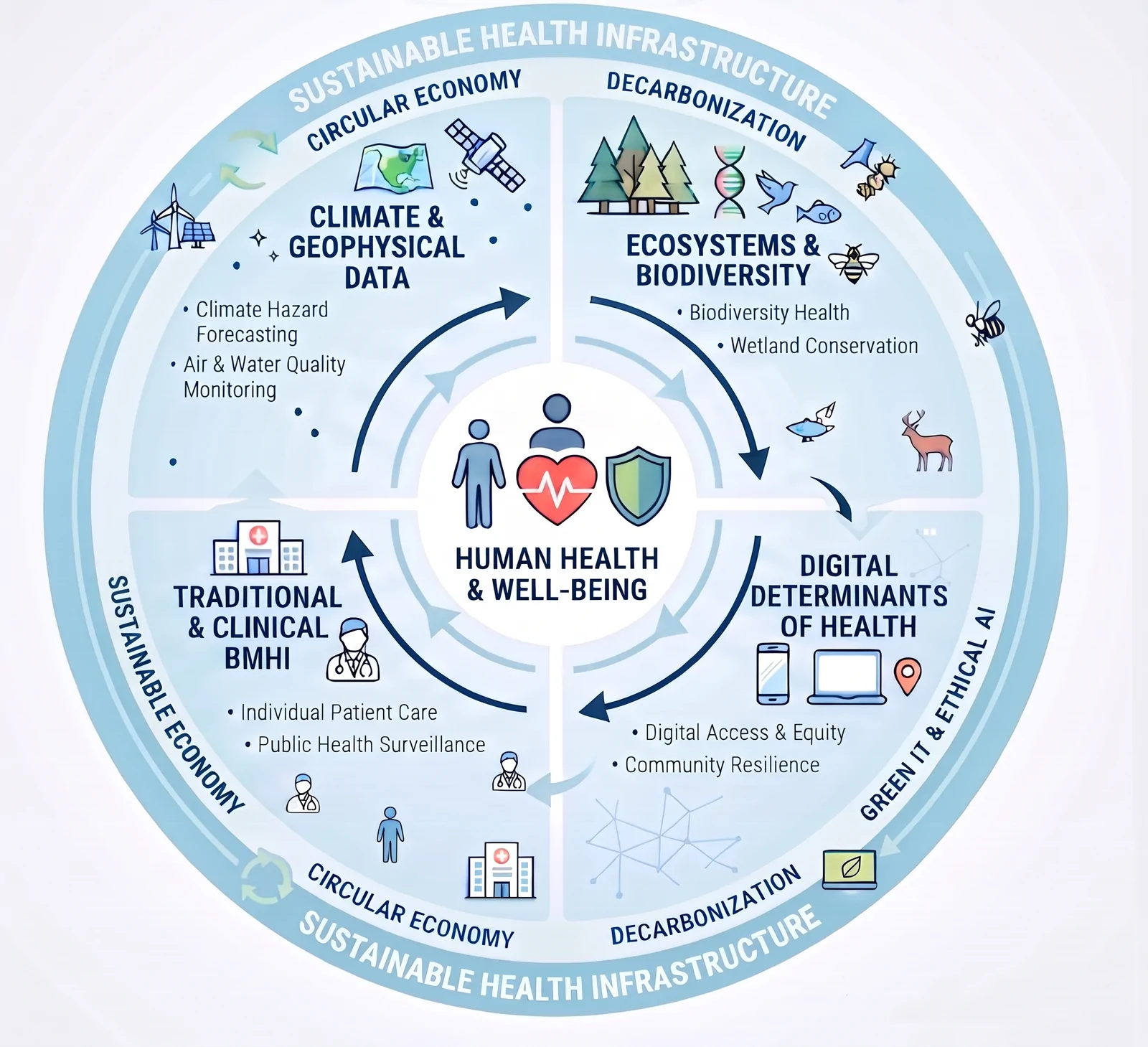
A representation of the integrated landscape of planetary health informatics. This diagram illustrates the conceptual expansion of BMHI beyond its traditional, human-centric, clinical focus. It maps the convergence of data streams from critical domains (climate and geophysical systems, ecosystems and biodiversity, and digital determinants of health) toward a central core of human health and well-being. The arrows indicate the necessary flow, integration, and feedback loops between these domains. Surrounding the entire system is the requirement for a sustainable health infrastructure (encompassing circular economy, decarbonization, and green IT), which ensures that the BMHI praxis itself does not destabilize the planetary systems it seeks to manage. BMHI: biomedical and health informatics.

## Call for Contributions

We invite the submission of manuscripts to the new *JMIR Medical Informatics* “Planetary Health Informatics” section. We welcome papers that address how health informatics can transcend the historical human-centricity of the discipline and bring information systems thinking to bear on the complex, interconnected health challenges of the Anthropocene. We are particularly interested in contributions that explore the integration of multispecies data, environmental indicators, and geophysical indicators with health information systems, digital tools for climate change adaptation, circular economies in digital health, and frameworks that foster interdisciplinary collaboration for a sustainable Anthropocene.

## Abbreviations

BMHI: biomedical and health informatics
ODH: One Digital Health
PHI: planetary health informatics
UN: United Nations
